# Feasibility of High‐Resolution Deuterium Metabolic Imaging of the Human Kidney Using Concentric Ring Trajectory Sampling at 7T

**DOI:** 10.1002/nbm.70139

**Published:** 2025-09-09

**Authors:** Fabian Niess, Bernhard Strasser, Lukas Hingerl, Johannes J. Kovarik, Viola Bader, Sabina Frese, Anna Duguid, Aaron Osburg, Eva Niess, Stanislav Motyka, Martin Krššák, Thomas Scherer, Wolfgang Bogner

**Affiliations:** ^1^ High‐Field MR Center, Department of Biomedical Imaging and Image‐Guided Therapy Medical University of Vienna Vienna Austria; ^2^ Clinical Division of Internal Medicine III Department of Nephrology and Dialysis Medical University of Vienna Vienna Austria; ^3^ Christian Doppler Laboratory for MR Imaging Biomarkers (BIOMAK), Department of Biomedical Imaging and Image‐guided Therapy Medical University of Vienna Vienna Austria; ^4^ Clinical Division of Internal Medicine III Department of Endocrinology and Metabolism Medical University of Vienna Vienna Austria

**Keywords:** 3D magnetic resonance spectroscopic imaging, deuterium labeled glucose, deuterium labeled water, deuterium metabolic imaging, DMI, kidney imaging

## Abstract

The human kidneys play a pivotal role in regulating blood pressure, water, and salt homeostasis, but assessment of renal function typically requires invasive methods. Deuterium metabolic imaging (DMI) is a novel, noninvasive technique for mapping tissue‐specific uptake and metabolism of deuterium‐labeled tracers. This study evaluates the feasibility of renal DMI at 7‐Tesla (7T) to track deuterium‐labeled tracers with high spatial and temporal resolution, aiming to establish a foundation for potential clinical applications in the noninvasive investigation of renal physiology and pathophysiology. Five healthy participants (3 m/2f) underwent renal DMI at 7T using MR spectroscopic imaging with concentric ring trajectory sampling. Two subjects participated in dynamic DMI experiments after oral administration of deuterium‐labeled water (D_2_O, 0.25, and 0.5 mL/kg) or glucose ([6,6′‐^2^H]‐Glc, 0.5 g/kg) following 12 h overnight fasting. Continuous glucose monitoring (CGM) was performed using FreeStyle Libre 3 and compared to renal ^2^H‐glucose levels. Three‐dimensional maps of ^2^H‐water and ^2^H‐glucose were acquired every ∼8.5 min at isotropic resolutions of ∼1.8 and ∼0.9 mL, respectively. Tensor Marchenko‐Pastur Principal Component Analysis (tMPPCA) was used for spectral denoising. Renal DMI successfully generated dynamic 3D maps of ^2^H‐water and ^2^H‐glucose with improved spatial resolution compared to previous studies. Following D_2_O ingestion, ^2^H‐water dynamics (0‐60 min) and steady‐state levels (> 90 min) were assessed. Following ^2^H‐glucose ingestion, renal ^2^H‐Glc concentrations peaked at 1.8 ± 1.0 mM on average over both kidneys, and overall dynamics aligned with interstitial glucose levels simultaneously assessed using CGM sensor. This study demonstrates the feasibility of dynamic renal DMI with improved spatial resolution to noninvasively map multiple ^2^H‐labeled tracers in the human kidney at 7T. Future improvements in signal mitigation and intravenous tracer administration could enhance its clinical utility, potentially aiding in the evaluation of metabolic effects of novel therapies like SGLT‐2 inhibitors for personalized treatment strategies.

## Graphical abstract

This study demonstrates the feasibility of renal deuterium metabolic imaging (DMI) at 7T to noninvasively map tracer dynamics in humans. Five healthy participants underwent DMI after ingestion of deuterium‐labeled water or glucose, achieving 3D maps with ∼0.9‐1.8‐mL resolution every 8.5 min. Renal ^2^H‐glucose dynamics correlated with continuous glucose monitoring, validating physiological relevance. These results establish renal DMI as a promising tool for investigating kidney metabolism, with potential clinical applications in therapy evaluation and personalized treatment strategies.

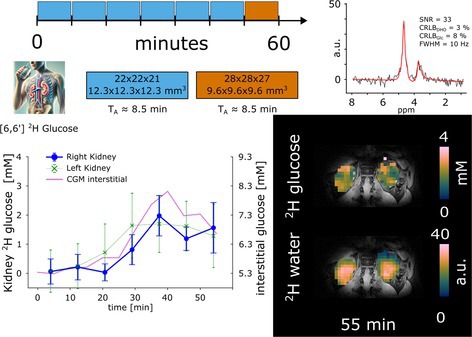

**Abbreviations**
CKD: chronic kidney disease
CRT: concentric ring trajectory
CSI: chemical shift imaging
DMI: deuterium metabolic imaging
FDG: fluorodeoxyglucose
FID: free induction decay
GFR: glomerular filtration rate
Glc: glucose
Glx: combined glutamine+glutamate
Lac: lactate
MRSI: magnetic resonance spectroscopic imaging
PET: positron emission tomography
SGLT: sodium glucose transporter
SNR: signal to noise ratio

## 1 Introduction

The human kidney is a vital organ with a high metabolic activity, playing a key role in various physiological functions such as blood filtration, hormone production, fluid and electrolyte balance, blood pressure regulation, acid–base and glucose homeostasis [1, 2]. Moreover, functional and metabolic alterations in the kidney are hallmarks of various pathological conditions, such as acute kidney injury [3], chronic kidney disease [4], diabetic nephropathy, and renal cell carcinoma [5]. To determine kidney function, the *glomerular filtration rate* (GFR) is considered the best overall index in health and disease. The gold standard method to measure GFR is by using plasma or urinary clearance of an exogenous filtration marker, such as iohexol, iothalamate, or alternatively chromium‐51 ethylenediamine tetra‐acetic acid [6]. However, the GFR test is cumbersome and impractical because of the difficulty in obtaining complete, accurately timed urine collections over a long time period. This is why, in clinics, in most cases GFR is estimated by using various formulas, such as the modification of Diet in Renal Disease (MDRD) or Chronic Kidney Disease Epidemiology collaboration equation (CKD‐EPI) using serum creatinine clearance. Alternatively, renal scintigraphy, a nuclear medicine technique, can be employed to assess renal perfusion and function and to estimate GFR using intravenously injected technetium‐based radiotracers [7]. Metabolic information, e.g., renal glucose uptake, can be assessed using [^18^F]‐fluorodeoxyglucose positron emission tomography ([^18^F]FDG‐PET), i.e., the clinical gold standard to image tissue‐specific glucose uptake [8]. However, [^18^F]FDG is excreted in the urine while, in the presence of normoglycemia, virtually all the filtered glucose is reabsorbed by sodium‐glucose cotransporters 1 and 2 (SGLTs) in the kidney. Consequently, the remaining detected radioactivity within the renal tubular space is mistaken as part of the glucose uptake by the surrounding cells [9]. Positive renal and cardiovascular effects during the treatment of patients with diabetes and chronic kidney disease (CKD) have been demonstrated using SGLT2‐inhibitors such as empagliflozin and dapagliflozin, which are novel, recently approved antihyperglycemic drugs, inhibiting renal glucose reabsorption and increasing urinary glucose release [10, 11]. Noninvasive and time‐resolved mapping of tissue‐specific renal glucose concentrations during an oral glucose challenge before and after the onset of SGLT2 therapy could help to better understand the metabolic effects of SGLT2‐inhibitor treatment.

In clinical practice, proton (^1^H) magnetic resonance imaging is used as a complementary fully noninvasive modality to assess anatomical and functional information, such as multi‐contrast high‐resolution structural images and maps of renal perfusion and oxygenation [12].

Deuterium metabolic imaging (DMI) is a novel MR technique to noninvasively image uptake and downstream metabolism of deuterium (^2^H)‐enriched substrates in the human body, using magnetic resonance spectroscopic imaging (MRSI) [13, 14, 15]. Recently, abdominal DMI was applied in humans at 3 and 7T, assessing dynamic DMI data from the liver, stomach, and kidney after oral administration of deuterium‐labeled glucose and water [16, 17, 18, 19, 20]. While multiple organs were covered in this study, only a few voxels [3, 4, 5, 6] were assigned to the kidney volume, with significant partial volume contamination, and not sufficient to spatially resolve differences across the kidney volume. Spatial resolution of conventional DMI methods is limited due to prolonged acquisition times of Cartesian phase‐encoding readout, rendering sub‐milliliter isotropic spatial resolution not feasible, especially for enlarged fields of view used in abdominal MR studies. Spatio‐spectral sampling can achieve significant acceleration factors compared to conventional phase‐encoding readout using different k‐space trajectories [21, 22, 23] and has recently been implemented for the DMI application [24, 25]. Hence, a non‐Cartesian concentric ring trajectory (CRT) readout for whole‐brain DMI at 7T enabled dynamic mapping of brain glucose metabolism with improved spatial resolution after oral administration of deuterium‐labeled glucose [25].

In this feasibility study, we have evaluated a fast CRT‐based ^2^H‐MRSI sequence for application in the kidney at 7T and demonstrate the feasibility of noninvasive dynamic assessment of multiple ^2^H‐labeled tracers simultaneously in both kidneys with higher spatial resolution than shown previously. Thereby, we establish a foundation for future clinical applications of DMI to study renal physiology and pathophysiology. Results of our study could pave the way for novel diagnostic and research applications, thereby enhancing our understanding of renal metabolism and function in health and disease and ultimately help to better understand the tissue specific metabolic consequences of novel antihyperglycemic drugs like SGLT‐2 inhibitors.

## 2 Material and Methods

### 2.1 Study Participants

Five healthy participants (BMI: 23± 3 kg/m^2^, age: 32± 7 years, 3 male/2 female) were included in the study after written informed consent was obtained. The study was approved by the local ethics committee of the Medical University of Vienna according to the guidelines of the Declaration of Helsinki. Optimization of sequence parameters and assessment of spectral quality was performed in phantom and in vivo using the natural abundance ^2^H water signal, while two volunteers underwent dynamic DMI measurements after oral administration of ^2^H‐labeled tracers. Two participants were scanned before and after oral uptake of ^2^H‐labeled water (D_2_O, heavy water, 0.25, and 0.5 mL/kg body weight mixed with ∼200 mL regular water) to assess the dynamics of D_2_O uptake within 60 min, and steady‐state concentrations after 90 min. The tracer was consumed after initial preparation measurements inside the scanner using an MR‐safe drinking bag. Similarly, one participant was scanned after overnight fasting in the morning after oral administration of ^2^H‐labeled glucose (0.5 g/kg body weight, [6,6′]‐^2^H‐Glc ≥99% purity, Cambridge Isotopes) dissolved in ∼200 mL water.

### 2.2 Continuous Glucose Monitoring

Before, during, and after the dynamic glucose experiment, continuous glucose monitoring of the upper arm's interstitial tissue (CGM) was performed using an MR‐safe monitoring device (Abbott Diabetes Care FreeStyle Libre 3). Intended use by the vendor does not officially include MR approval, but the sensor was extensively tested for MR safety in a recent study [26], and the local ethics committee approved the use of the device for investigational purposes in the current study.

### 2.3 DMI Protocol

**FIGURE 1:**
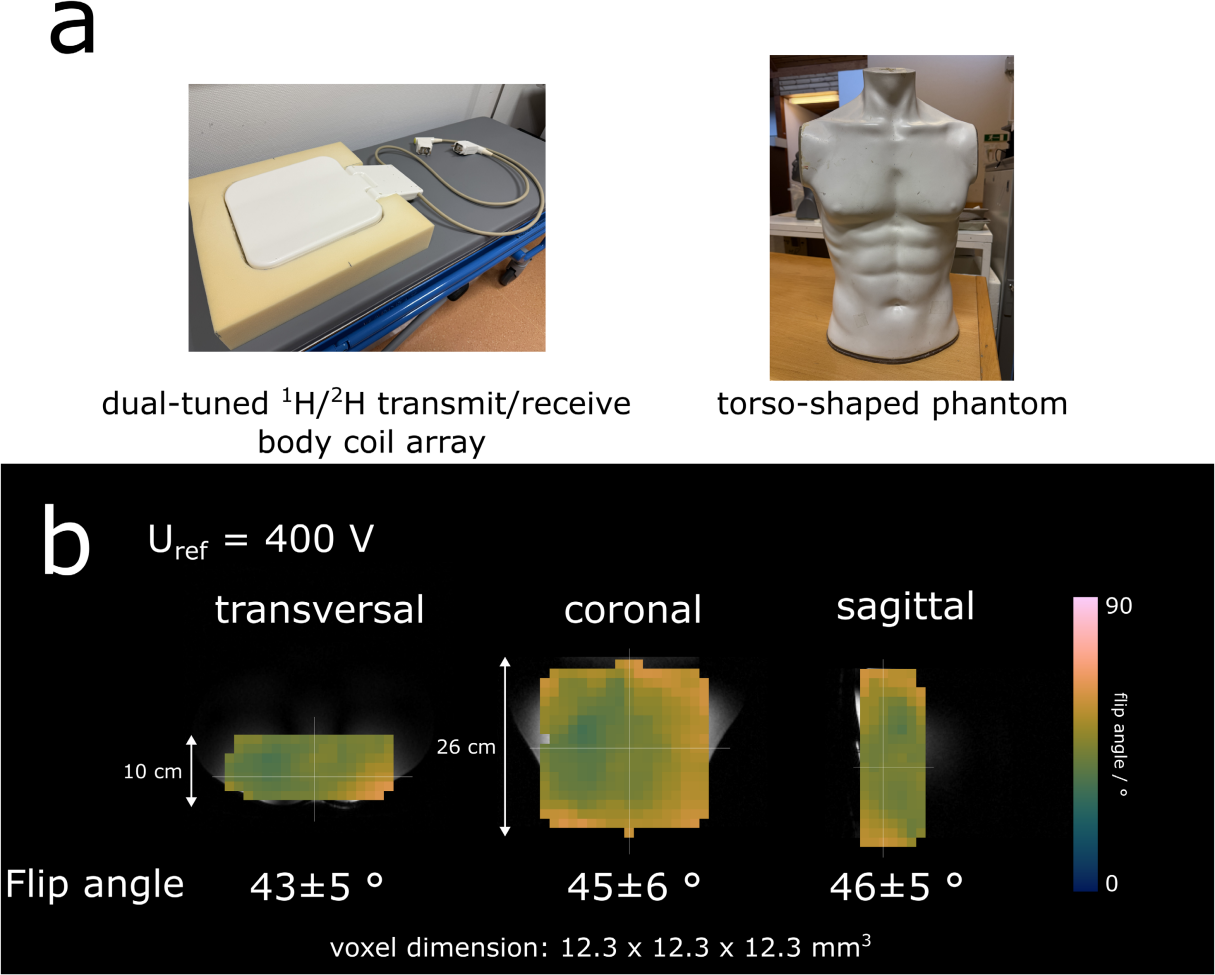
(a) Experimental setup illustrating the dual‐tuned ^2^H/^1^H body coil array inserted in the plastic foam and the torso‐shaped phantom mimicking real in vivo tissue properties. (b) Orthogonal B_1_^+^ maps acquired from the torso‐shaped phantom using the double‐angle method. Averaged flip angles are given across representative transversal, coronal, and sagittal slices.

All measurements were performed on a human 7T whole‐body MR system (Magnetom dot Plus, Siemens, Healthineers, Erlangen) using a dual‐tuned (^2^H/^1^H) body coil array [one ^2^H transmit channel (~27 × 27 cm): two ^2^H receive channels: each loop (~17 × 17 cm) and one butterfly (~25 × 15 cm)], (Stark Contrasts MRI Coils Research, Germany). The coil was placed in a foamed‐plastic insert posterior to the participants' lower back, while the participants were lying supine on the patient bed (Figure 1a). The acquisition of ^2^H signals was natively supported by Siemens' hardware/software implementation. No hardware or software modifications were required.

Initial preparation scans included gradient‐echo‐based tri‐planar localizer images followed by 3D gradient‐echo acquisitions for anatomical imaging using the following parameters: FOV: 340 × 340 × 224 mm^3^, matrix: 256× 256× 160, T_R_ = 3.4 ms, T_E_ = 1.38 ms, T_A_ = 1:55 min. Correct coil positioning for a reliable assessment of Kidney DMI data was validated using the ^1^H localizer scans. Shimming was performed using the standard 3D shimming routine supplied by the vendor (i.e., “DESS” followed by “GRE‐BREAST”) with the adjust volume placed in a way that covered both kidneys.

DMI was performed using a previously developed ^2^H‐FID MRSI sequence [22] using 3D density‐weighted CRT readout [27, 28, 29, 30] with ∼1.8 mL (matrix: 22 × 22 × 21) and ∼0.9 mL (matrix: 28 × 28 × 27) nominal spatial resolution and the following parameters: non‐localized rectangular excitation pulse (pulse duration: 2 ms), FOV: 270 × 270 × 260 mm^3^, TR = 290 ms, acquisition delay = 2.0 ms, N_circles_ = 47, readout bandwidth = 380 Hz, number of spectral points = 96, T_A_ = 8:35 min. To test the feasibility of slice‐selective excitation, the sequence was modified to incorporate a Hamming‐weighted sinc pulse (TBWP = 2.13) and an asymmetric sinc pulse (TBWP = 3.1) and phantom measurements were performed using a spherical water phantom (diameter = 160 mm). Detailed information about sequence parameters is provided in Table S1 [31].

### 2.4 Segmentation

Manual segmentation of the kidney was performed on high resolution anatomical ^1^H 3D GRE images using ITK‐SNAP [32]. High resolution segmentation masks were down‐sampled to MRSI grid size using MINC tools (MINC tools, v2.0, McConnell Brain Imaging Center, Montreal, QC, Canada).

### 2.5 B_1_^+^ Estimation

B_1_^+^ estimation of the ^2^H‐channels was performed in a separate experiment using a Torso mannequin‐shaped phantom filled with tissue‐like gel mimicking in vivo properties [33] (electrical conductivity σ = 0.60 S/m, relative permittivity ε = 62) (Figure 1a). The exact recipe of the phantom can be found in [34]. B_1_^+^ maps were measured via the Double‐Angle Method [35] with a T_R_ = 2500 ms and a T_A_ = 30 min (N_circles_ = 29) for each reference voltage (U_ref_ = 400 V and 800 V).

To validate the correct B_1_^+^ estimation 3D DMI maps of ^2^H natural abundance water, the scans were repeated in one participant using three different reference voltages (720, 800 and 840 V). Voxel‐wise signal‐to‐noise ratios (SNR) were calculated using the amplitudes of ^2^H natural abundance water and the standard deviation of the noise in a region ∼100 Hz off‐resonant. Mean and standard deviation of voxel‐wise SNR values were calculated over both kidneys using segmentation masks.

### 2.6 Dynamic and Steady‐State DMI of D_2_O Uptake

Following 2D and 3D anatomical ^1^H imaging scans and one reference DMI acquisition (detecting natural abundance ^2^H water) the D_2_O + H_2_O mixture was orally administered either inside the scanner to dynamically assess renal D_2_O uptake within 60 min or outside of the scanner for the steady‐state measurement after 90 min. The dynamic experiment employed eight consecutive DMI acquisitions using 1.8‐mL nominal isotropic spatial resolution, with a total scan duration of ∼80 min. The steady‐state measurement consisted of four dynamic DMI acquisitions using 1.8 mL (three time points) and 0.9 mL (one time point) nominal spatial resolution, with an overall measurement time of ∼55 min.

### 2.7 Dynamic DMI of ^2^H—Glc Uptake

After initial preparation scans including localizer images, 3D anatomical ^1^H imaging, and B_0_ shimming, two DMI reference scans of the natural abundance ^2^H water signal were acquired using 1.8 mL (matrix: 22 × 22 × 21) and 0.9 mL (matrix: 28 × 28 × 27) nominal spatial resolution, respectively. Following oral consumption of the deuterium‐labeled glucose inside the scanner bore, a dynamic DMI acquisition was performed over the course of ∼60 min using both resolution protocols (six repetitions with 1.8 mL and one repetition with 0.9 mL spatial resolution).

### 2.8 Data Post Processing and Metabolite Quantification

Image reconstruction and spectral fitting of ^2^H‐labeled metabolite resonances was performed offline using a custom‐built post processing pipeline (MATLAB R2021b, LCModel v6.3, Python3.12). Data reconstruction was performed using non‐Cartesian three‐dimensional discrete Fourier Transformation [28, 29]. Coil combination was performed using Whitened Singular Value Decomposition (WSVD) [36, 37]. To mitigate the effect of decreased SNR due to increased spatial resolution, spectral denoising was performed using tensor Marchenko‐Pastur Principal Component Analysis (tMPPCA) [38, 39]. For spectral fitting of DMI data, a basis set was simulated using NMR‐Scope‐B [40, 41] including ^2^H resonances of “natural abundance” deuterium water (4.8 ppm) and Glc (3.9 ppm). Quantification results with Cramer‐Rao Lower Bounds (CRLBs) > 50% were excluded from further analysis. Voxel‐wise SNR was calculated similar to B_1_^+^ estimation measurements, while linewidths, given as Full Width at Half Max (FWHM), and CRLBs were estimated using LCModel.

### 2.9 Concentration Estimation

Fitted concentrations of ^2^H‐labeled water in 3D DMI maps are shown using arbitrary units.

Voxel‐wise concentration estimation (in mM) was performed for 3D DMI maps of ^2^H‐Glc for all time points using internal referencing to ^2^H natural abundance water. To the best of our knowledge, so far, no relaxation times for ^2^H resonances in the human kidney have been reported at 7T. Therefore, we assumed an average kidney water content of 80%, comparable to that of the healthy human brain as well as similar longitudinal (T_1_) and transversal (T_2_) relaxation times of ^2^H water and ^2^H Glc as recently reported for brain tissue, i.e., (T_1water_ = 335 ms, T_1Glc_ = 70 ms; T_2water_ = 36 ms, T_2Glc_ = 36 ms) [42, 43]. The number of ^2^H atoms per molecule was accounted for and concentration estimation was performed as proposed in [44, 45].

Kidney voxels exceeding 5 mM glucose concentration, equaling a maximum increase of 90 mg/dL, were excluded from the analysis to avoid overestimation of glucose values due to potential contamination of strong glucose signals from the stomach or small intestines.

### 2.10 Statistical Analysis

The Friedman test was used to compare SNR values of all voxels in the kidney between three different reference voltages. A paired t‐test was used to estimate differences in data quality between left and right kidney volume with a statistical significance threshold of *p* < 0.05 [46]. The Benjamini and Hochberg method was used to adjust *p*‐values for false discovery rate correction of multiple testing. Statistical tests were performed using Python 3.12 (www.python.org, packages: scipy.stats). For metabolic maps, the “batlow” color map was used as recommended in [47].

## 3 Results

### 3.1 B_1_^+^ Estimation

**FIGURE 2:**
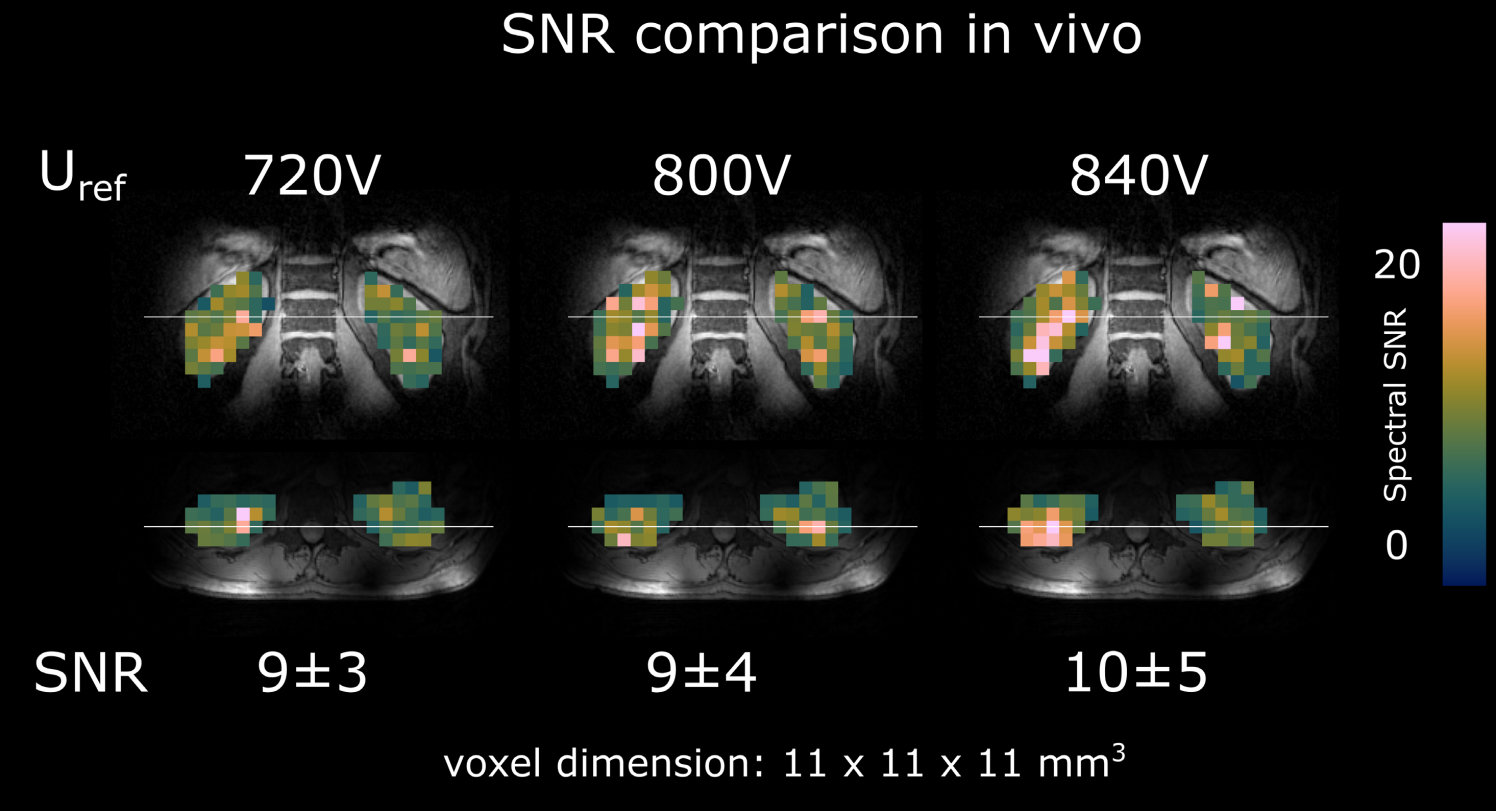
Comparison of spectral ^2^H‐water signal‐to‐noise ratios (SNR) across the entire kidney volume for different reference voltages (720, 800, and 840 V). No significant differences were observed, indicating that an~90° excitation flip angle is reached at ~800 V.

Using 400 V reference voltage for ^2^H signal transmission, the double angle method for rapid B_1_^+^ mapping yielded, on average, 43 ± 5°, 45 ± 6°, and 46 ± 5° flip angles for representative transversal, coronal, and sagittal slices of the torso‐shaped phantom, respectively (Figure 1b). To validate ^2^H B_1_^+^ estimation results from the torso‐shaped phantom and approximate the needed reference voltage for a 90° flip angle, in vivo measurements were performed using 720, 800, and 840 V reference voltage, and the average spectral SNR over the kidney volume was compared. No significant differences (*p* = 0.11) were found between different reference voltages, with 9 ± 3, 9 ± 4, and 10 ± 5 for 720, 800, and 840 V, respectively (Figure 2).

### 3.2 Data Quality Natural Abundance ^2^H Water In Vivo

**FIGURE 3:**
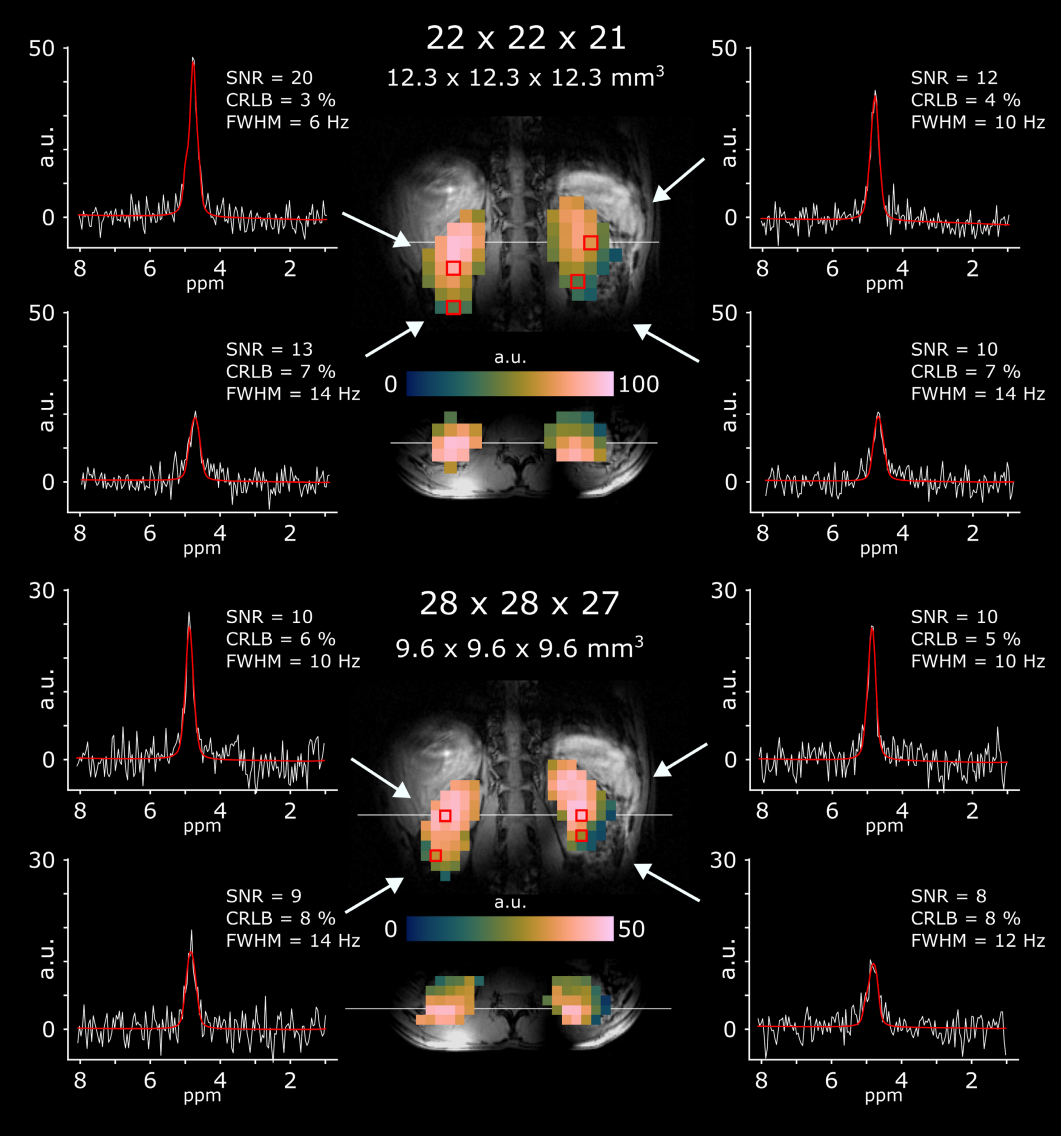
Representative spectra of natural abundance ^2^H‐labeled water in kidney voxels for two spatial resolutions (1.8 and 0.9 mL nominal voxel volume). Raw spectra (white), LCmodel fits (red) and signal quality metrics, including ^2^H‐water linewidths (FWHM), Cramer‐Rao Lower Bounds (CRLBs), and signal‐to‐noise ratio (SNR), are shown in one representative participant.

Signals from natural abundant ^2^H‐labeled water in the human kidney were reliably detected using both 1.9 mL (12.3 × 12.3 × 12.3 mm^3^) and 0.8 mL (9.6 × 9.6 × 9.6 mm^3^) nominal spatial resolution. Estimated SNR, fitting precision (i.e., CRLBs) and spectral linewidths (i.e., FWHM) averaged over both kidney and over all subjects were 10 ± 3, 8 ± 2%, and 14 ± 1 Hz, respectively. No differences were observed between voxels of the left and right kidney across all participants for SNR (*p* = 0.53), CRLBs (*p* = 0.4) and linewidths (*p* = 0.22). Representative sample spectra and respective fit results for both spatial resolutions from one participant are shown in Figure 3. Individual results of all participants are shown in Table 1.

**TABLE 1:** Summary of participant characteristics and imaging parameters including body mass index (BMI), signal‐to‐noise ratio (SNR), linewidth (full‐width at half maximum, FWHM), Cramer‐Rao Lower Bounds (CRLB) of water, global FWHM of water, voxel volume, excitation flip angle, and repetition time (TR). B_1_ estimation was performed in subject No. 1 which explains why *T*_R_ and nominal voxel size and flip angle differs slightly. Dynamic DMI measurement were performed in subject number 4 and 5 with oral uptake of ^2^H‐water and ^2^H‐glucose, respectively. Flip angle was chosen using the optimal Ernst angle for the target metabolites (only water, or combined water + glucose). Data reflect individual values for the study participants.

*Subjects*	BMI [kg/m^2^]	Sex	SNR	FWHM [Hz]	CRLB [%]	^2^H shim [Hz]	Nominal voxel volume [ml]	Flip angle [°]	T_R_ [ms]
*1*	24	m	7 ± 4	13 ± 4	11 ± 6	72	1,3	90	350
*2*	22	f	12 ± 5	14 ± 5	6 ± 2	70	1,8	67	290
*3*	21	f	15 ± 7	15 ± 5	6 ± 5	60	1,8	67	290
*4*	21	m	8 ± 4	15 ± 4	9 ± 4	60	1,8	67	290
*5*	26	m	9 ± 4	13 ± 4	9 ± 4	69	1,8	90	290

For natural abundance ^2^H‐labeled water scans, tMPPCA denoising increased the spectral SNR from 8 ± 2 to 10 ± 3 (*p* = 0.004), while CRLBs decreased from 11 ± 3% to 8 ± 2% (*p* = 0.017) on average for both kidney volumes across all participants. No line broadening was observed due to spectral denoising; i.e., similar FWHM values (*p* = 0.1) were obtained with 13 ± 1 Hz and 14 ± 1 Hz before and after denoising, respectively.

For time‐resolved data from one participant acquired during the dynamic DMI experiment after oral administration of ^2^H‐Glc, improved denoising resulted in a significant increase in SNR from 7 ± 0.2 to 14 ± 1 (*p* < 0.001), averaged across all kidney voxels at a nominal isotropic spatial resolution of 1.8 mL. Furthermore, CRLBs for fitting of ^2^H‐labeled resonances decreased on average across time points from 12 ± 0.4% to 7 ± 0.5% (*p* < 0.001) for water and from 23 ± 3% to 14 ± 2% (*p* < 0.001) for glucose (last 3 time points), while similar linewidths were observed (*p* = 0.9). Representative spectra before and after tMPPCA denoising from a single time point 55 min after oral administration of ^2^H‐Glc are shown in Figure S1.

### 3.3 Dynamic and Steady‐State DMI of D_2_O Uptake

**FIGURE 4:**
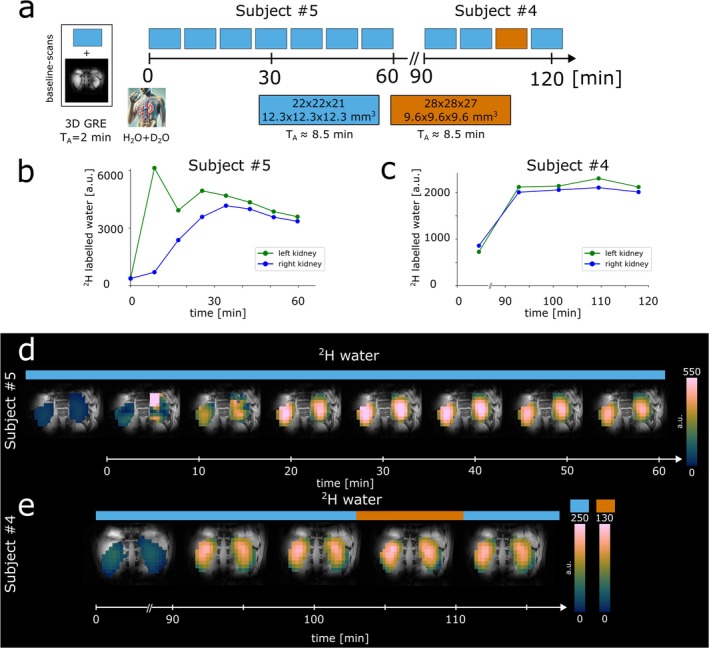
Illustration of the DMI protocol used for ^2^H‐water administration (a), summed time courses of the ^2^H‐water signal across the left and right kidney (b,c) and time courses of representative coronal slices of ^2^H‐labeled water signals for both dynamic and steady‐state experiments with different nominal spatial resolutions (1.8, blue; 0.9 mL, orange) (d,e).

Experimental setup, averaged time courses, and coronal DMI maps of the human kidney for ^2^H‐labeled water are illustrated for each time point and both dynamic and steady‐state DMI experiments in Figure 4a. To compare ^2^H‐water maps between two different spatial resolutions employed during the steady‐state experiment (1.8 mL: 1st—3rd and 5th time points, 0.9 mL: 4th time point), the signal sum over all voxels across the 3D kidney volume was calculated instead of using the mean and standard deviation. During the dynamic DMI assessment, strong signal amplitudes were observed in the left kidney volume directly after D_2_O consumption, followed by a more homogenous distribution after ∼10 min, see Figure 4b. A maximum ^2^H‐labeled signal increase of 10‐fold compared to baseline was observed 30 min after D_2_O consumption before gradually decreasing until the end of the dynamic experiment. For the steady‐state experiment, a 2.8‐fold signal increase of ^2^H‐labeled water was observed in both kidneys 94 min after oral uptake of D_2_O and stayed relatively stable for approximately 35 min with a coefficient of variation (COV) of 3.7% (Figure 4c).

### 3.4 Dynamic DMI of ^2^H‐Glc Uptake

**FIGURE 5:**
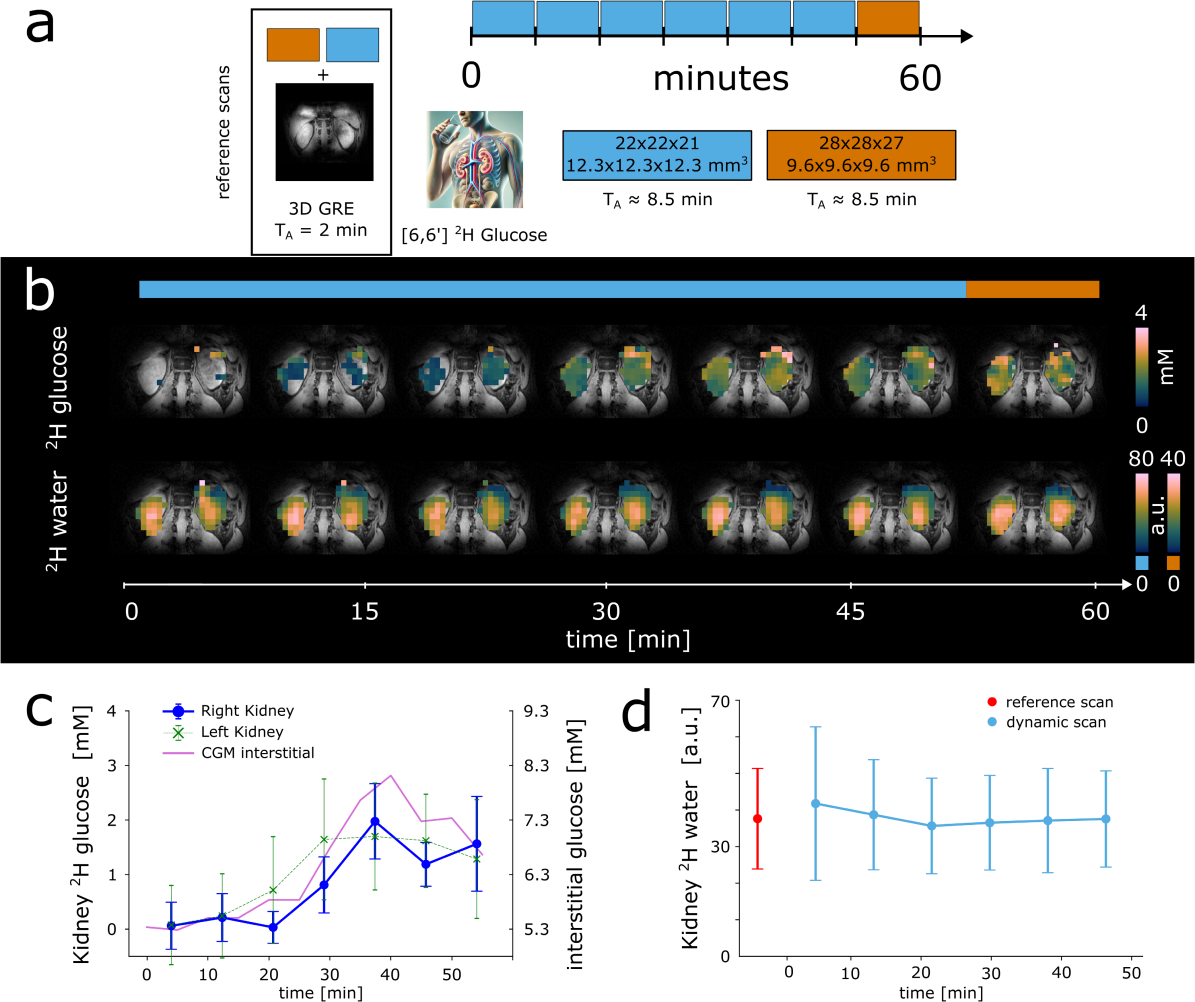
Illustration of the dynamic DMI protocol used for ^2^H‐glucose administration (a). Following initial anatomical 2D and 3D imaging scans dynamic deuterium metabolic imaging (DMI) maps of ^2^H‐labeled glucose from the human kidney after oral ^2^H‐Glc administration are displayed. Time courses of representative coronal maps for ^2^H‐labeled glucose concentration estimation in mM and ^2^H‐labeled water signals (a.u.) with 1.8 and 0.9 mL nominal spatial resolution are shown (b). Time courses of the interstitial and averaged ^2^H‐labeled glucose concentrations in the left and right kidney (c) and water signals averaged over both kidneys are also displayed (d).

Coronal DMI maps of the human kidney for ^2^H‐labeled glucose for two spatial resolutions, e.g., 1.8 mL (1st—6th time points) and 0.9 mL nominal spatial resolution (7th time point) are also illustrated (Figure 5a,b). The time courses of averaged renal glucose concentrations together with interstitial glucose levels are shown in Figure 5c. Time courses of averaged water signals are illustrated in Figure 5d. CRLBs for spectral fits of ^2^H‐labeled water and glucose are shown in Figure S2.

37 min after oral uptake of ^2^H‐labeled glucose, the detected ^2^H‐Glc levels (over both kidneys) reached 1.95 ± 1.0 mM with 2.1 ± 0.67 mM in the right and 1.82 ± 0.96 mM in the left kidney (Figure 5c). Relative to baseline interstitial glucose levels of 5.27 mM at the beginning of the DMI scan, comparable glucose dynamics were detected using continuous glucose measurement (CGM) reaching a maximum increase of 2.78 mM (absolute: 8.05 mM) 40 min after glucose consumption before gradually decreasing toward baseline levels (Figure S3).

For the scans with 1.8‐mL nominal isotropic voxel volume, the average CRLBs for fitting of ^2^H‐glucose over the kidney volume and all time points were not significantly different between left and right kidney (*p* = 0.66) with 20 ± 6% and 21 ± 8%, respectively, while lower CRLBs were obtained for ^2^H water in the right kidney (5 ± 0.1%) compared to the left kidney 7 ± 0.35% (*p* < 0.001). Significantly higher SNR (24 ± 1 vs. 16 ± 1, *p* < 0.001) and smaller linewidths (10 ± 1 Hz vs. 14 ± 1 Hz, *p* = 0.0033) were observed in the right kidney compared to the left kidney over all time points.

After oral consumption of ^2^H‐Glc, high signal concentrations were observed anterior to both kidney volumes, presumably originating from the stomach and small intestines, which is illustrated in unmasked ^2^H‐Glc DMI maps in Figure S4.

To avoid potential signal contamination from strong signals anterior to the kidneys, 3D DMI using slice‐selective excitation was tested using two additional RF pulse shapes (Half‐Sinc and asymmetric Sinc) on a water phantom to illustrate the feasibility (Figure S5).

## 4 Discussion

In this study we propose a fully noninvasive technique to map the dynamics of ^2^H‐labeled tracers in the human kidney. We demonstrated the feasibility of our MR method for robust time‐resolved imaging of different ^2^H‐labeled substrates in the human kidney with improved spatial resolution compared to what has been reported in other comparable abdominal DMI studies at 3 and 7T of a different scope, which focused on the coverage of multiple abdominal organs [16, 17, 18, 19, 20]. However, the prolonged sampling duration of conventional phase encoding sequences generally limits achievable spatial resolution beyond 15‐mL nominal isotropic resolution for dynamic experiments. This study did not aim for a direct comparison between high‐resolution renal DMI acquired using our proposed CRT sampling scheme and lower‐resolution DMI maps acquired using conventional Cartesian phase encoding schemes. Effects of the spatial resolution, i.e., partial volume effects and point spread function, have been reported in a previous DMI study by our group in a different organ [25]. In this study, three‐dimensional DMI maps of ^2^H‐labeled water and glucose were dynamically acquired in both kidneys using ^2^H‐MRSI with CRT readout, achieving significant acceleration and nominal voxel volumes of ~1.8 and ~0.9 mL.

Optimal sequence parameters were validated using natural abundance ^2^H‐labeled water signals of the kidney tissue without tracer administration, which could be mapped with good signal quality and fit precision.

D_2_O accumulation in the tissue of the human kidney was assessed dynamically for 60 min after D_2_O consumption and in a steady‐state experiment 90 min after uptake using 3D DMI with 1.8 and 0.9‐mL nominal isotropic resolution. The increase of ^2^H‐labeled water signals compared to natural abundance ^2^H‐water levels was comparable in both kidneys. Strong signal amplitudes in the left kidney volume at the beginning of the dynamic experiment are presumably caused by signal contamination originating from the stomach or smaller intestines. However, the disappearance of strong ^2^H‐labeled water signals over time is in good agreement with stomach clearance kinetics recently proposed in a comparable study [18]. Elevated ^2^H‐water concentrations remained relatively stable throughout the steady‐state measurement (∼34 min) indicating a period of fluid homeostasis, as ^2^H‐labeled water is actively reabsorbed into the bloodstream. However, further measurements validating this claim, including the assessment of osmolality parameters from blood or urine samples, would be required, which was not the aim of this study. Different concentrations of D_2_O were applied for the dynamic and the steady‐state experiment to investigate dose effects on the renal ^2^H‐labeled water signals. Although an apparent linear increase was observable, the small sample size and differences in the experimental timing prevent further analysis. Steady‐state or dynamic mapping of D_2_O uptake with increased spatial resolution could potentially be applied in future studies to provide tumor contrast between benign, malignant, and healthy renal masses [18], as reported in preclinical studies [48], and potentially detect effects of early response to radiation or chemotherapy [49]. Compared to more conventional ^1^H MRI‐based approaches to measure renal perfusion, such as arterial spin labeling, dynamic contrast‐enhanced imaging, and diffusion‐weighted imaging with intravoxel incoherent motion analysis [50], the administration of D_2_O can provide similar information. This can be assessed indirectly using ^1^H MRI [51] or directly using DMI [52] with lower spatial resolution. However, DMI potentially allows the administration of multiple deuterium‐labeled tracers simultaneously and can provide additional complementary information. Time‐resolved mapping of the kidney's ^2^H‐Glc uptake was successfully performed using our proposed method, which involved assessing 3D DMI maps with nominal isotropic voxel volumes of 1.8 and 0.9 mL every ∼8.5 min. Similar levels of ^2^H‐glucose, but higher variability, were observed across the left kidney compared to the right kidney. Although differences in spectral linewidth and fitting precision were observed between the left and right kidney, presumably due to imperfect B_0_ shimming, higher variability in ^2^H‐glucose levels of the left kidney is presumably caused by residual contamination from strong ^2^H‐glucose accumulation inside the stomach and small intestines anterior to the kidney.

Oral administration of ^2^H‐labeled substrates is a limitation of abdominal DMI studies in general, as strong signals from the stomach and smaller intestines potentially contaminate other regions of interest. To remove outliers and unreasonably high glucose values, an individual threshold of 5 mM glucose was chosen, i.e., 90 mg/dL glucose increase relative to baseline levels. Therefore, 2% of kidney voxels were excluded, i.e., 20 out of 857. This is in agreement with glucose data acquired in the interstitial tissue using the CGM sensor, which showed a glucose increase well below this level. Another potential way to minimize signal contamination from outside of the kidney is using slice‐selective RF excitation instead of non‐localized excitation, which was investigated using phantom measurements. However, due to the low gyromagnetic ratio of ^2^H, slice selection requires very high gradient amplitudes, which ultimately limits the minimum achievable slice thickness to ∼4–5 cm. To improve the excitation slice profile, Hamming‐weighted sinc and asymmetrically shaped sinc RF pulses with prolonged pulse durations are required to stay within specific absorption rate constraints. However, this increases the echo time and reduces the respective RF excitation bandwidth, which leads to increased chemical shift displacement errors. Intravenous injection of ^2^H‐labeled glucose would entirely avoid signal contamination of the stomach and small intestines, and presumably could be the preferred choice over oral application for abdominal DMI studies. This could potentially be used in future studies.

One of the limitations of DMI applications is that only the fraction of ^2^H‐labeled glucose is detected, while unlabeled glucose baseline levels prior to oral tracer administration remain invisible. Therefore, precise estimation of fractions of labeled/unlabeled glucose concentration would require time‐resolved blood sampling and estimation of the deuterium enrichment. As this exceeds the scope of this feasibility study, we decided not to include invasive blood sampling and assumed that endogenous glucose production during hyperglycemia of healthy individuals is negligible. We therefore hypothesized that increasing overall glucose levels are mainly caused by orally administered ^2^H‐labeled glucose and correlate with ^2^H‐labeled glucose dynamics detected in the kidney via DMI. Hence, increasing interstitial glucose levels simultaneously detected using the CGM sensor were comparable with ^2^H‐labeled glucose values detected in the kidney. If the renal threshold is not exceeded, glucose levels in the kidney should mimic blood glucose concentrations [1], but in our data, a small mismatch of ∼33% between maximum ^2^H‐glucose levels of the right kidney detected via DMI and the increase in interstitial glucose levels relative to baseline was observed. This could be presumably caused by inaccurate assumptions of water content (assumed 80% on average) and ^2^H relaxation times that are necessary for concentration estimation using DMI data. While similar longitudinal relaxation times of D_2_O have been reported in rabbit kidneys at 4.7T [53], no relaxation times of D_2_O and glucose have been reported in the human kidney at 7T. However, simultaneous CGM measurements inside the MR scanner could potentially be used in future metabolic studies featuring an oral glucose challenge as a normalization reference. While interstitial glucose levels do not directly reflect blood glucose concentrations, they are highly correlated, and CGM sensors are widely used in diabetes care.

Although the kidney is a metabolically active organ, we were not able to detect downstream metabolites of the aerobic and anaerobic glucose utilization, e.g., Glx and lactate. Lactate concentrations at the papillary tip were predicted to be 5.85 mM [54]; however, the respective volume is presumably too small to significantly contribute to the overall signal detected using our proposed method.

The dimensions of the ^1^H‐channels of the dual‐tuned surface coil array were smaller than those of the ^2^H‐channels at 17 × 15 cm^2^ and 27 × 27 cm^2^, respectively, but yielded sufficiently high‐quality localizer images, shimming, and high‐resolution GRE images to locate the kidney position and perform simple manual segmentation of the kidney shape. More sophisticated segmentation of different tissue types of the human kidney, e.g., cortex and medulla, would require ^1^H images with higher quality and contrast, which are not feasible using our current transceiver body coil array and represent a limitation of this study. Abdominal ^1^H MRI is strongly affected by B_0_ and B_1_ field inhomogeneities and motion artifacts, especially at 7T. To improve tissue segmentation, high‐resolution images with improved quality could be obtained at lower field strengths, e.g., at 3T, followed by co‐registration to images acquired using the current setup. As differentiation between tissue types was not the main focus of this study, and the spatial resolution of the DMI data is still a major limiting factor to resolve regional differences on a small scale, we used ^1^H data acquired from the same session.

The sensitive volume of the ^2^H‐channels of the dual‐tuned transceiver RF coil was sufficient to obtain good signal quality of ^2^H‐labeled resonances from the kidney tissue, allowing up to sub‐milliliter isotropic resolution. The relatively big coil dimensions of the ^2^H transmit channel (27 × 27 cm^2^) lead to an excitation profile (B_1_^+^) with excellent homogeneity across almost the entire sensitive coil volume, reaching at least 10 cm deep into the abdomen, but require high reference voltages (∼800 V, i.e., U_Pulse_ = 200 V for rectangular pulse with 2 ms duration) and consequently high energy deposition. As B_1_^+^ estimation in vivo showed no significant differences between reference voltages of 720–840 V, we chose 720 V for all measurements to remain within the specific absorption rate constraints for a T_R_ of 290 ms.

Low‐rank based spectral denoising, e.g., using tMPPCA, has been shown to reduce measurement uncertainty [27, 55]. Although SNR and CRLBs were significantly improved, without affecting the line shape needs to be interpreted with caution. Thus, we chose a relatively high CRLB threshold of 50% to remove outliers. Especially for low SNR spectra, which are expected at the beginning of a dynamic ^2^H‐Glc DMI experiment, it is not recommended to exclude data points according to strict SNR and/or CRLB thresholds [56, 57]. The performance of low‐rank approaches for spectral denoising benefits from data redundancy, also along the dynamic dimension. This was also observed in our DMI data when comparing the improvements to 3D datasets of natural abundance ^2^H‐water of a single time point. This supports the notion that DMI measurements should be performed and evaluated dynamically, as data quality benefits from improved denoising. Additionally, time‐resolved DMI scans can be directly correlated with functional or metabolic activity.

Inherently low SNR remains the biggest challenge when performing DMI studies. This ultimately limits the achievable spatial resolution. A further increase in spatial resolution could be technically achieved without prolonging the scan duration of 8.5 min for each time point. In fact, the minimum achievable scan duration for this matrix size is ∼1–2 min when only one average is obtained. Thus, depending on the type of experiment and SNR, which is tracer and concentration dependent, our method allows for flexible adjustments of the spatial and temporal resolution.

^2^H‐labeling is not limited to water and glucose and can be applied to many other metabolically active substances, which are potentially interesting for investigation of kidney function and metabolism, e.g., choline [53, 58, 59, 60] or the ketone Beta‐hydroxybutyrate (BHB), which is involved in renal energy metabolism, and so far has only been investigated in preclinical DMI studies in animals [61]. Additionally, DMI is not limited to administration of a single tracer at a time, and in principle, multiple tracers could be used simultaneously.

DMI of the kidney could offer unique and clinically relevant insight into metabolic changes of the tissue and could provide complementary information to analyze and better understand effects of, e.g., novel SGLT2‐inhibiting drugs blocking glucose re‐absorption in the proximal tubule, such as empagliflozin and dapagliflozin [10, 11], which is not feasible using [^18^F]FDG‐PET. As the kinetics of renal glucose concentrations during an oral glucose challenge in patients undergoing SGLT2‐inhibition treatment are not entirely clear, dynamic DMI with increased spatial resolution could enhance the understanding of renal metabolism, potentially optimize treatment strategies for diabetes and CKD, and aid the development of new interventions for renal and cardiovascular health.

## 5 Conclusion

In conclusion, we presented the technical feasibility for fully noninvasive mapping of different ^2^H‐labeled tracer dynamics over time with high spatial resolution in the human kidney using DMI at 7T.

This work established a foundation for future clinical applications of DMI in renal physiology and pathology to potentially image regional differences in ^2^H‐labeled tracer uptake rates in participants with impaired kidney function and monitor metabolic changes during treatment response. The gained insights could pave the way for novel diagnostic and research applications, enhancing our understanding of renal metabolism and function in health and disease.

## Author Contributions

**Fabian Niess:** conceptualization, methodology, validation, formal analysis, investigation, data curation, visualization, writing – original draft, visualization. **Bernhard Strasser:** software, methodology, validation, writing – review and editing. **Lukas Hingerl:** software, methodology, writing – review and editing. **Johannes J. Kovarik:** conceptualization, funding, investigation, writing – review and editing. **Viola Bader:** investigation, writing – review and editing. **Sabina Frese:** investigation, writing – review and editing. **Anna Duguid:** investigation, writing – review and editing. **Aaron Osburg:** investigation, writing – review and editing. **Eva Niess:** investigation, visualization, writing – review and editing. **Stanislav Motyka:** investigation, writing – review and editing. **Martin Krššák:** investigation, writing – review and editing. **Thomas Scherer:** investigation, writing – review and editing. **Wolfgang Bogner:** conceptualization, methodology, resources, supervision, project administration, writing – review and editing.

All authors reviewed and approved the final version of the manuscript.

## Conflicts of Interest

The authors declare no conflicts of interest.

## Supporting information


**Table S1:** Minimum Reporting Standards for in vivo MR Spectroscopy. Note. – Parameters 7T DMI, CRLB = Cramér‐Rao lower bounds; FID = free induction decay; CRT = concentric ring trajectory; FOV = field of view; FWHM = full‐width‐at‐half‐maximum; Glx = Glutamate+Glutamine; Glc = Glucose; SNR = signal‐to‐noise ratio; VOI = volume of interest.
**Figure S1:** Representative sample raw spectra, spectral fit and residue are shown before and after tensor Marchenko‐Pastur Principal Component Analysis (tMPPCA) denoising for a single time point of one participant during the dynamic DMI experiment (45 min after oral glucose administration). Signal quality metrics, including linewidths (FWHM) of water and Cramer‐Rao Lower Bounds (CRLBs) of water and glucose and signal‐to‐noise ratio (SNR), are given for each voxel.
**Figure S2:** CRLB time courses for spectral fits of ^2^H‐labled water and glucose averaged across the entire kidney volume.
**Figure S3:** Extended continuous glucose monitoring (CGM) measurements from the interstitial fluid of the upper arm. Data capture glucose concentration dynamics 1 h before, during, and 1 h after oral glucose administration.
**Figure S4:** Unmasked deuterium metabolic imaging (DMI) maps of ^2^H‐glucose illustrating signal contamination from high glucose concentrations presumably in the stomach and small intestines anterior to the kidney. This should emphasize potential challenges in kidney‐specific glucose quantification.
**Figure S5:** Phantom study illustrating feasibility of slice‐selective RF excitation using half‐sinc and asymmetric sinc RF pulses. The images demonstrate the slice profiles achievable with different pulse shapes, potentially reducing signal contamination in targeted regions, while increasing energy deposition, chemical shift displacement error and prolonging acquisition delays.

## Data Availability

Data generated by postprocessing (i.e., metabolic maps, LCModel basis sets, script files for data plotting) are available from the corresponding author on reasonable request for research purposes only. Data generated by postprocessing methods (i.e., metabolic maps, LCModel basis sets, Python and MATLAB script files for data analysis and plotting) are available from the corresponding author on reasonable request for research purposes only.
