# Supplementary material for: Feasibility of High‐Resolution Deuterium Metabolic Imaging of the Human Kidney Using Concentric Ring Trajectory Sampling at 7T
Source: NMR Biomed. 2025 Sep 9;38(10):e70139. doi: 10.1002/nbm.70139 (PMC12418914; doi:10.1002/nbm.70139)
Supplement: Supplementary file 1 — Table S1: Minimum Reporting Standards for in vivo MR Spectroscopy. Note. – Parameters 7T DMI, CRLB = Cramér‐Rao lower bounds; FID = free induction decay; CRT = concentric ring trajectory; FOV = field of view; FWHM = full‐width‐at‐half‐maximum; Glx = Glutamate+Glutamine; Glc = Glucose; SNR = signal‐to‐noise ratio; VOI = volume of interest. Figure S1: Representative sample raw spectra, spectral fit and residue are shown before and after tensor Marchenko‐Pastur Principal Component Analysis (tMPPCA) denoising for a single time point of one participant during the dynamic DMI experiment (45 min after oral glucose administration). Signal quality metrics, including linewidths (FWHM) of water and Cramer‐Rao Lower Bounds (CRLBs) of water and glucose and signal‐to‐noise ratio (SNR), are given for each voxel. Figure S2: CRLB time courses for spectral fits of 2H‐labled water and glucose averaged across the entire kidney volume. Figure S3: Extended continuous glucose monitoring (CGM) measurements from the interstitial fluid of the upper arm. Data capture glucose concentration dynamics 1 h before, during, and 1 h after oral glucose administration. Figure S4: Unmasked deuterium metabolic imaging (DMI) maps of 2H‐glucose illustrating signal contamination from high glucose concentrations presumably in the stomach and small intestines anterior to the kidney. This should emphasize potential challenges in kidney‐specific glucose quantification. Figure S5: Phantom study illustrating feasibility of slice‐selective RF excitation using half‐sinc and asymmetric sinc RF pulses. The images demonstrate the slice profiles achievable with different pulse shapes, potentially reducing signal contamination in targeted regions, while increasing energy deposition, chemical shift displacement error and prolonging acquisition delays. [file NBM-38-e70139-s001.docx]

| *Minimum Reporting Standards in MR Spectroscopy checklist (according to Lin et al. NMR Biomed 2021)* | |
| --- | --- |
| **1. Hardware** |  |
| *a. Field strength [T]* | 7T |
| *b. Manufacturer* | Siemens |
| *c. Model (software version if available)* | Magnetom dot Plus |
| *d. RF coils: nuclei (transmit/ receive), number of channels, type, body part* | ^2^H/^1^H dual tuned body coil array, transmit/receive, 1 channel transmit / 2 channels receive, (Stark Contrast MRI Coils Research, Germany) |
| *e. Additional hardware* | N/A |
| **2. Acquisition** |  |
| *a. Pulse sequence* | 3D FID-acquire density-weighted concentric ring trajectory (CRT) MRSI |
| *b. Volume of Interest (VOI) locations* | kidney, unlocalized excitation using rectangular RF pulse with 67 or 86° flip angle |
| *c. Nominal VOI size [cm^3^, mm^3^]* | FOV 250x250x240 mm^3^ |
| *d. Repetition Time (TR), Echo Time (TE) [ms, s]* | TR = 290 ms / 2 ms acquisition delay |
| *e. Total number of Excitations or acquisitions per spectrum* | 47 circles 8.5 min acquisition time |
| *In time series for kinetic studies* | N/A |
| *i.         Number of Averaged spectra (NA) per time-point* | N/A |
| *ii.       Averaging method (e.g. block-wise or moving average)* | N/A |
| *iii.      Total number of spectra (acquired / in time-series)* | N/A |
| *f. Additional sequence parameters (spectral width in Hz, number of spectral points, frequency offsets); If STEAM: Mixing Time TM; If MRSI: 2D or 3D, FOV in all directions, matrix size, acceleration factors* | CRT MRSI: BW: 380 Hz, 96 spectral points, 22x22x21 or 28x28x27 |
| *g. Water Suppression Method* | No water suppression |
| *h. Shimming Method, reference peak, and thresholds for “acceptance of shim” chosen* | Standard DESS+GRE-BREAST shim + manual adjustment, ^1^H water peak < 100 Hz, global ^2^H water peak < 70 Hz Region: both kidneys |
| *i. Triggering or motion correction method (respiratory, peripheral, cardiac triggering, incl. device used and delays)* | - |
| **3. Data analysis methods and outputs** |  |
| *a. Analysis software* | LCModel 6.3-1 |
| *b. Processing steps deviating from quoted reference or product* | N/A |
| *c. Output measure (e.g. absolute concentration, institutional units, ratio)* | concentration estimation in mM and a.u. |
| *d. Quantification references and assumptions, fitting model assumptions* | Simulated in NMRScope-B |
| **4. Data Quality** |  |
| *a. Reported variables (SNR, Linewidth (with reference peaks))* | SNR was calculated using voxel-wise signal maximum of water / sd of noise 200 Hz off-center |
| *b. Data exclusion criteria* | CRLBs > 50 % for water, Glc, |
| *c. Quality measures of postprocessing Model fitting (e.g. CRLB, goodness of fit, SD of residual)* | CRLB |
| *d. Sample Spectrum* | See Figure 3 and Supplementary Figure 1 |

**Supplementary Table 1:**

Minimum Reporting Standards for in vivo MR Spectroscopy

Note. – Parameters 7T DMI**,** CRLB = Cramér-Rao lower bounds; FID = free induction decay; CRT = concentric ring trajectory; FOV = field of view; FWHM = full-width-at-half-maximum; Glx = Glutamate+Glutamine; Glc = Glucose; SNR = signal-to-noise ratio; VOI = volume of interest.


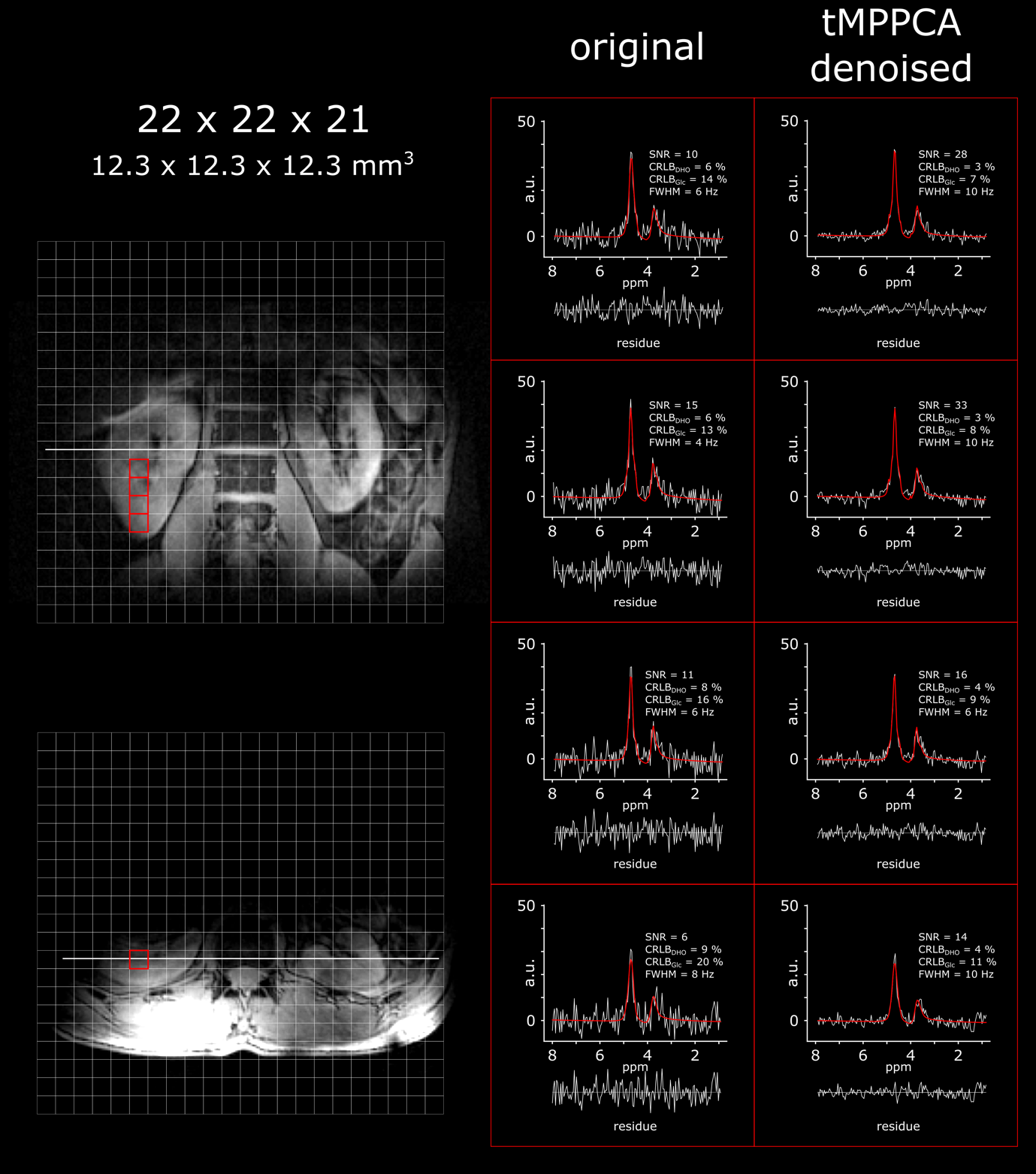


**Supplementary Figure 1:**

Representative sample raw spectra, spectral fit and residue are shown before and after tensor Marchenko-Pastur Principal Component Analysis (tMPPCA) denoising for a single time point of one participant during the dynamic DMI experiment (45 minutes after oral glucose administration). Signal quality metrics, including linewidths (FWHM) of water and Cramer-Rao Lower Bounds (CRLBs) of water and glucose and signal-to-noise ratio (SNR), are given for each voxel.


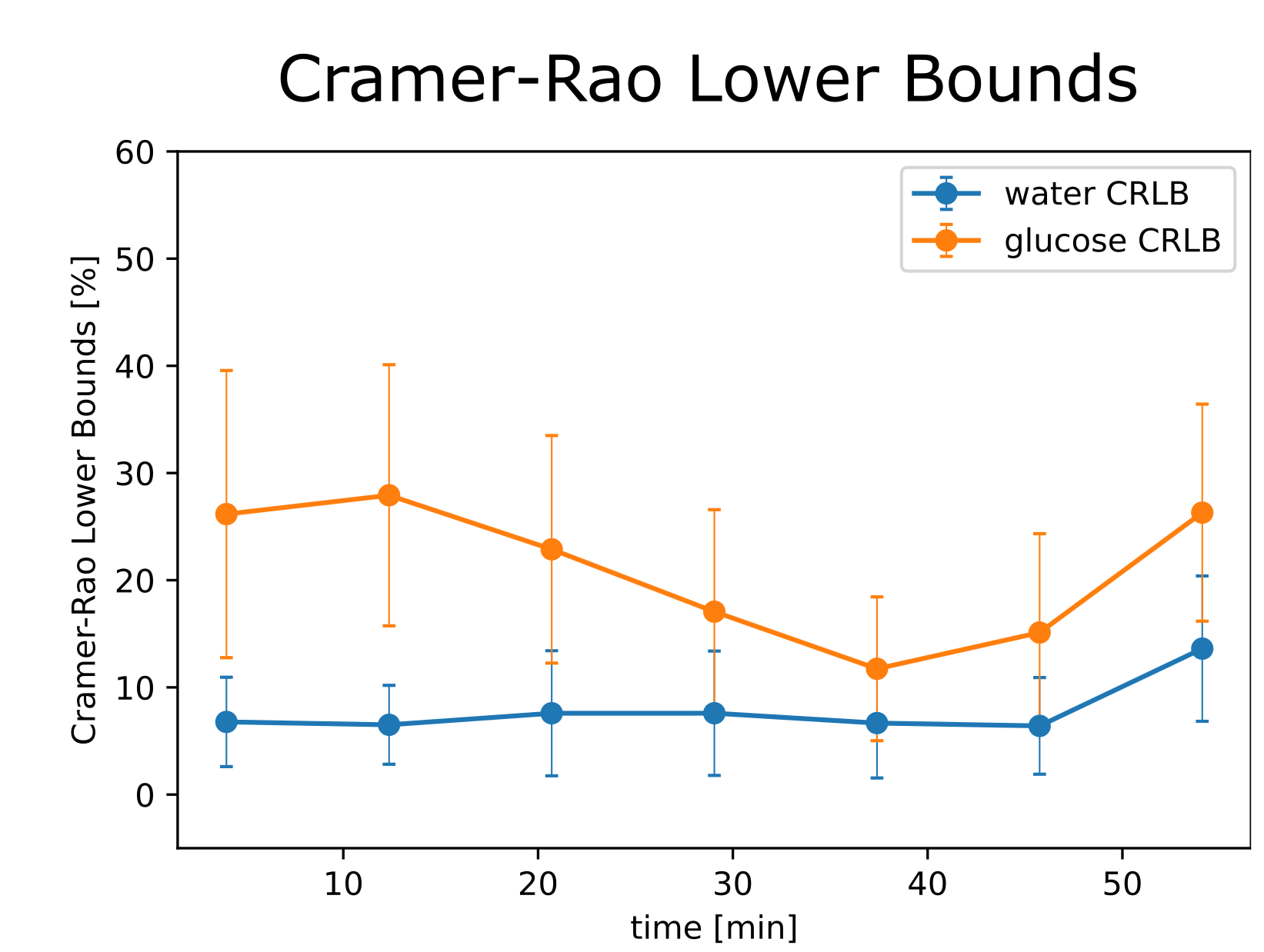


**Supplementary Figure 2:**

CRLB time courses for spectral fits of ^2^H-labled water and glucose averaged across the entire kidney volume.

**
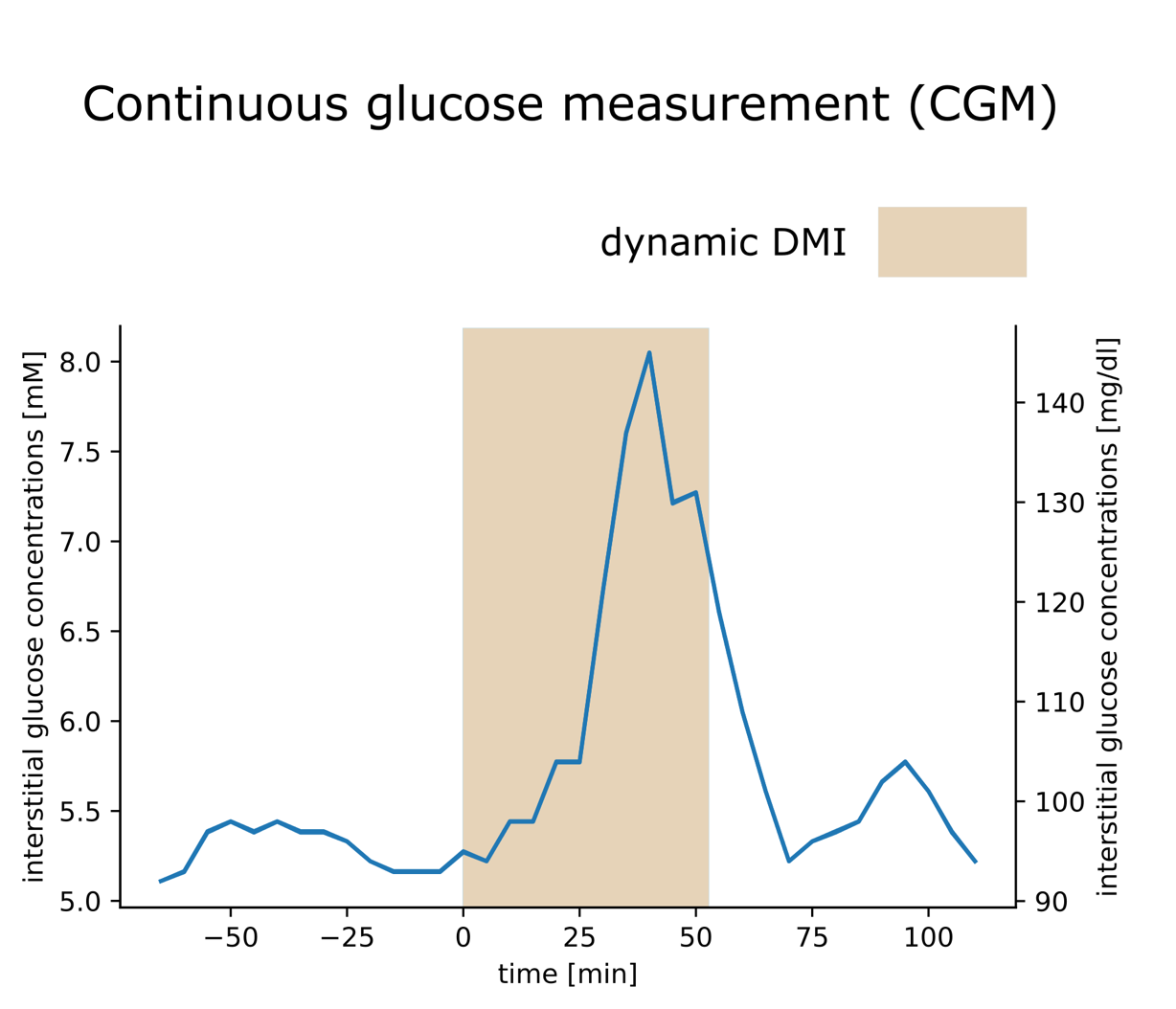
 Supplementary Figure 3:**

Extended continuous glucose monitoring (CGM) measurements from the interstitial fluid of the upper arm. Data capture glucose concentration dynamics one hour before, during, and one hour after oral glucose administration.


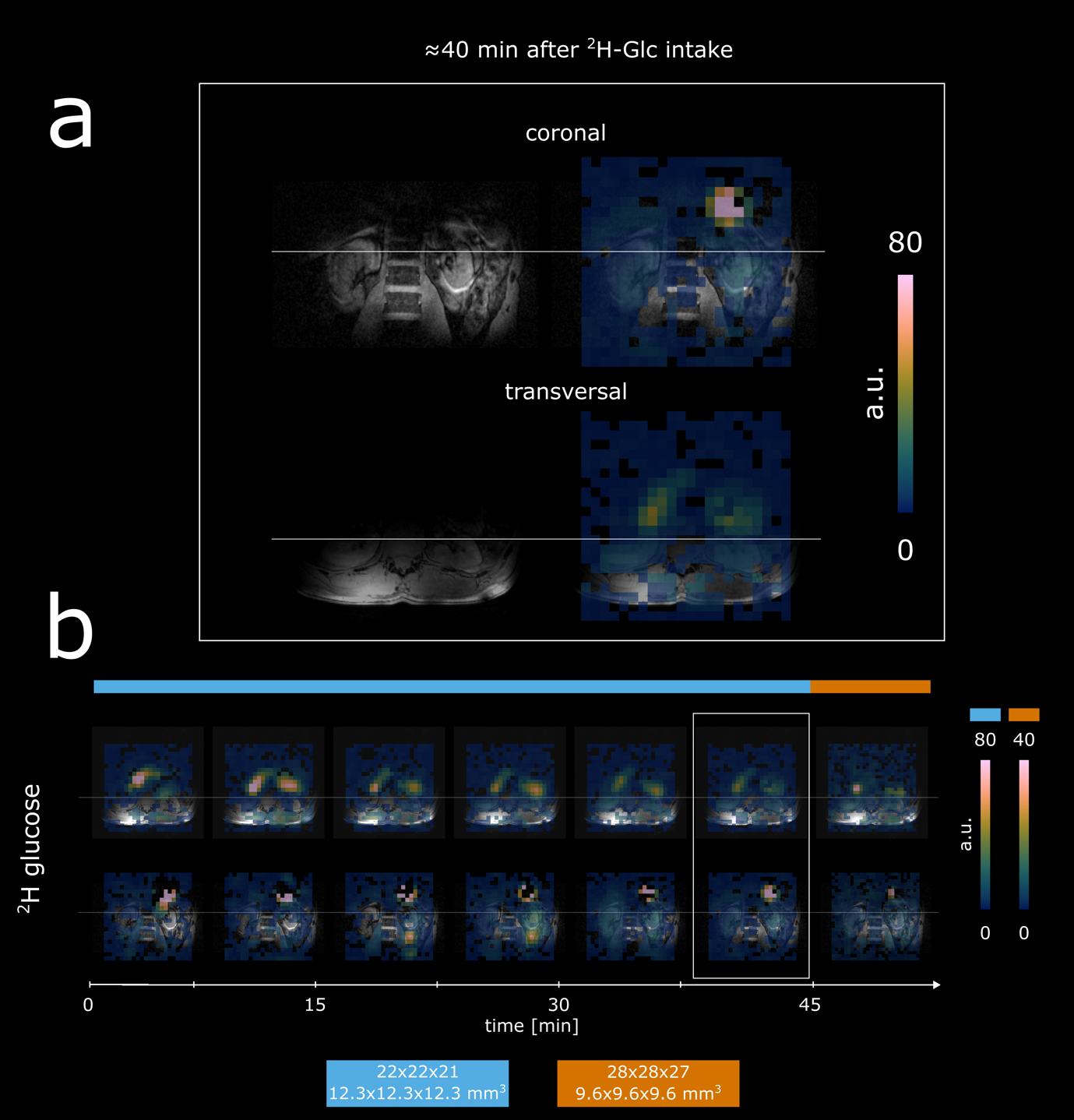


**Supplementary Figure 4:**

Unmasked deuterium metabolic imaging (DMI) maps of ^2^H-glucose illustrating signal contamination from high glucose concentrations presumably in the stomach and small intestines anterior to the kidney. This should emphasize potential challenges in kidney-specific glucose quantification.


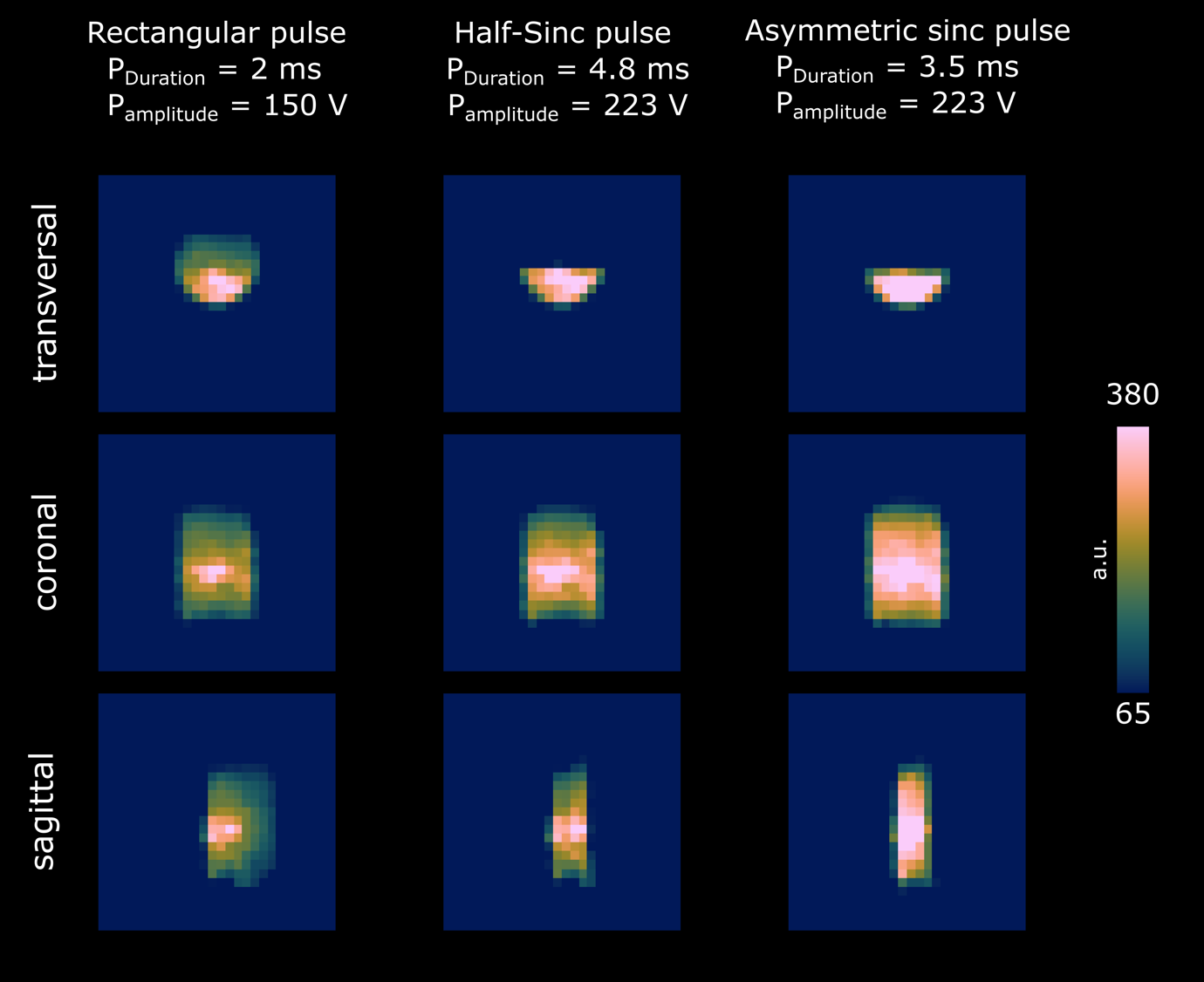


**Supplementary Figure 5:**

Phantom study illustrating feasibility of slice-selective RF excitation using half-sinc and asymmetric sinc RF pulses. The images demonstrate the slice profiles achievable with different pulse shapes, potentially reducing signal contamination in targeted regions, while increasing energy deposition, chemical shift displacement error and prolonging acquisition delays.
